# Evaluation of the antimalarial activity and toxicity of Mahanil-Tang-Thong formulation and its plant ingredients

**DOI:** 10.1186/s12906-022-03531-2

**Published:** 2022-02-27

**Authors:** Prapaporn Chaniad, Arisara Phuwajaroanpong, Tachpon Techarang, Natharinee Horata, Arnon Chukaew, Chuchard Punsawad

**Affiliations:** 1 Department of Medical Sciences, School of Medicine, Walailak University, Nakhon Si Thammarat, 80160 Thailand; 2 Faculty of Medical Technology, Huachiew Chalermprakiet University, Samutprakan, 10540 Thailand; 3 Chemistry Department, Faculty of Science and Technology, Suratthani Rajabhat University, Surat Thani, 84100 Thailand

**Keywords:** Antimalarial activity, Toxicity, Malaria, Medicinal plants, Mahanil-Tang-Thong formulation

## Abstract

**Background:**

Novel potent antimalarial agents are urgently needed to overcome the problem of drug-resistant malaria. Herbal treatments are of interest because plants are the source of many pharmaceutical compounds. The Mahanil-Tang-Thong formulation is a Thai herbal formulation in the national list of essential medicines and is used for the treatment of fever. Therefore, this study aimed to evaluate the antimalarial activity of medicinal plants in the Mahanil-Tang-Thong formulation.

**Methods:**

Nine medicinal plant ingredients of the Mahanil-Tang-Thong formulation were used in this study. Aqueous and ethanolic extracts of all the plants were analyzed for their phytochemical constituents. All the extracts were used to investigate the in vitro antimalarial activity against *Plasmodium falciparum* K1 (chloroquine-resistant strain) by using the lactate dehydrogenase (pLDH) method and cytotoxicity in Vero cells by using the 3-(4,5-dimethylthiazol2-yl)-2,5-diphenyltetrazolium bromide (MTT) assay. Additionally, an extract with potent in vitro antimalarial activity and no toxicity was selected to determine the in vivo antimalarial activity with Peters’ 4-day suppressive test against the *Plasmodium berghei* ANKA strain. Acute toxicity was evaluated in mice for 14 days after the administration of a single oral dose of 2000 mg/kg.

**Results:**

This study revealed that ethanolic extracts of *Sapindus rarak* DC., *Tectona grandis* L.f., *Myristica fragrans* Houtt. and *Dracaena loureiri* Gagnep. exhibited potent antimalarial activity, with half-maximal inhibitory concentration (IC_50_) values of 2.46, 3.21, 8.87 and 10.47 μg/ml, respectively, while the ethanolic of the formulation exhibited moderate activity with an IC_50_ value of 37.63 μg/ml and its aqueous extract had no activity (IC_50_ = 100.49 μg/ml). According to the in vitro study, the ethanolic wood extract of *M. fragrans* was selected for further investigation in an in vivo mouse model. *M. fragrans* extract at doses of 200, 400, and 600 mg/kg body weight produced a dose-dependent reduction in parasitemia by 8.59, 31.00, and 52.58%, respectively. No toxic effects were observed at a single oral dose of 2000 mg/kg body weight.

**Conclusion:**

This study demonstrates that *M. fragrans* is a potential candidate for the development of antimalarial agents.

## Background

Malaria drug resistance is a major problem in malaria chemotherapy control and has affected mortality and morbidity rates [1, 2]. Drug resistance to chloroquine (CQ) and sulfadoxine-pyrimethamine (SP) has developed and spread widely [3–5]. In 1971, artemisinin was first isolated from the plant *Artemisia annua*, a medicinal plant that has commonly been used in traditional Chinese medicine; it has efficacious effects against all multidrug resistant forms of *Plasmodium falciparum* [6]. Artemisinin and its derivatives are potent and rapidly acting antimalarial drugs that cause a fast decline in parasitemia during the first day of treatment [7]. Artemisinin-based combination therapies (ACTs) are now recommended for malaria treatment, but artemisinin resistance in *Plasmodium falciparum* has been reported in Southeast Asia [8]. Antimalarial drug resistance manifests as spontaneous mutations or gene duplications that decrease drug susceptibility [9]. Resistance to CQ in parasites requires the *P. falciparum* chloroquine resistance transporter (*Pf*crt) gene, which leads to parasite death by the accumulation of drugs in parasite food vacuoles [10]. Antifolate drugs, including SP, inhibit the *P. falciparum* dihydropteroate synthetase (*Pf*DHPS) and *P. falciparum* bifunctional dihydrofolate reductase-thymidylate synthase (*Pf*DHFR) enzymes, leading to the inhibition of folate biosynthesis [1, 11]. For artemisinin resistance, the point mutation in the K13 propeller gene has been reported as a key determinant in *P. falciparum* [12]. Due to this drug resistance crisis, new therapeutics are needed. Herbal treatment is an intriguing choice because plants are the source of many pharmaceutical compounds [13]. In Thailand, many traditional medicine products derived from plants have been recognized in the national list of essential medicines [14]. For example, the Mahanil-Tang-Thong formulation is a Thai herbal formulation in the national list of essential medicines and comprises nine herbal plants, namely, *Calamus axillaris* Becc., *Dracaena loureiri* Gagnep., *Entada rheedii* Spreng., *Myristica fragrans* Houtt., *Pogostemon cablin* (Blanco) Benth., *Sapindus rarak* DC., *Spondias pinnata* (L.f)*.* Kurz, *Tectona grandis* L.f., and *Tiliacora triandra* (Colebr.) Diels. This formulation possesses antipyretic effects and is used for the treatment of fever [14]. However, there is no Thai traditional formulation specifically for treating malaria. Based on the clinical symptoms of malaria, all patients present with an acute febrile illness, including basic symptoms of fever, chills and headache [15]; therefore, this study hypothesized that Mahanil-Tang-Thong formulation and its plant ingredients might have potential application in antimalarial treatments.

For the Mahanil-Tang-Thong formulation, there have been no reports of antimalarial activity. Although some medicinal plants in this formulation have been investigated for antimalarial activity, the vast majority are limited to in vitro models [16]. Because the search for substances with potent antimalarial activity and low toxicity both in vitro and in animal models is of great importance, it is necessary to discover good candidates for antimalarial drugs. Therefore, this study aimed to evaluate the antimalarial activity of the Mahanil-Tang-Thong formulation and its plant ingredients and further investigated the antimalarial effects of plants that possess good candidates in animal models. In addition, a toxicity study was performed to determine any poisoning effects.

## Materials and methods

### Selection of medicinal plants

Nine medicinal plant ingredients of the Mahanil-Tang-Thong formulation obtained from a Thai traditional drug store in Nakhon Si Thammarat were used in this study. The collection of plant materials followed the relevant guidelines and regulations of The Plant Varieties Protection, Department of Agriculture, Ministry of Agriculture and Cooperatives, Thailand. Plant identification was performed by Assoc. Prof. Tanomjit Supavita, the School of Pharmacy, Walailak University. Voucher specimens were deposited at the Department of Medical Sciences, School of Medicine, Walailak University, Thailand (Table 1).

**Table 1:** List of medicinal plants of the Mahanil-Tang-Thong formulation used in this study

Plant species	Family	Plant part	Voucher number
*Calamus axillaris Becc.*	Arecaceae	Stem	SMD 198012003
*Dracaena loureiri* Gagnep.	Dracaenaceae	Wood	SMD 096001007
*Entada rheedii* Spreng.	Leguminosae - Mimosoidaea	Seed kernel	SMD 147008003
*Myristica fragrans* Houtt.	Myristicaceae	Wood	SMD 177001003
*Pogostemon cablin* (Blanco) Benth.	Lamiaceae	Aerial part	SMD 142031002
*Sapindus rarak* DC.	Sapindaceae	Fruit	SMD 241019001
*Spondias pinnata* (L.f.) Kurz	Anacardiaceae	Seed	SMD 011018005
*Tectona grandis* L.f.	Lamiaceae	Wood	SMD 142036001
*Tiliacora triandra* (Colebr.) Diels	Menispermaceae	Leaf	SMD 170014001

### Preparation of crude extract

Dried plants were washed with tap water and dried in a hot air oven **(**Memmert, Model; SFE600, Schwabach, Germany**)** at 60 °C**.** Then, the plants were pulverized by using a grinder (Taizhou Jincheng Pharmaceutical Machinery Co., Ltd., Model; SF, Jiangsu, China) and separated into two parts for aqueous and ethanolic extraction. Aqueous extraction was performed as described by Chaniad et al. [17]. Briefly, plant powder was mixed in distilled water at a proportion of 1:10 under reflux. Then, the marc was re-extracted, and the combined extracts were filtered with gauze followed by Whatman filter paper number 1 (Whatman, Buckinghamshire, England). A rotary evaporator (Rotavapor, Buchi, China) was used to concentrate the extract under vacuum at 50 °C, and the extract was further lyophilized to dryness with a freeze-dryer (Christ Gamma 2–16 LSCplus, Germany) to obtain the aqueous extract. The ethanolic extract was prepared by soaking 60 g of plant powder in 600 ml of ethanol solvent for 72 h**.** This procedure was repeated once, and then, the combined ethanolic extract from the first and second extractions was filtered and evaporated as described above**.** Finally, both dried aqueous and ethanolic extracts were weighed to calculate the percent yield and stored in screwcap containers at 4 °C until use to prevent contamination.

### Phytochemical analysis

Eighteen crude extracts from two different extraction solvents (water and ethanol) were screened for phytochemical constituents using the protocol described by Ngbolua and Malar et al. [18, 19] for the determination of flavonoids, terpenoids, alkaloids, tannins, anthraquinone, cardiac glycosides, saponins and coumarins.

### In vitro assay

#### Cultivation of *Plasmodium falciparum* blood stage

The *Plasmodium falciparum* K1 strain (chloroquine-resistant strain) was obtained from Dr. Rapatbhorn Patrapuvich, Department of Drug Research Unit for Malaria, Faculty of Tropical Medicine, Mahidol University, Thailand. The cultivation protocol followed methods described in Trager and Jensen with some modifications [20]. Briefly, incomplete RPMI 1640 culture medium (Gibco/Invitrogen, Montreal, Canada) was buffered with 2 mg/ml sodium bicarbonate, 4.8 mg/ml HEPES (Himedia, Mumbai, India), 10 μg/ml hypoxanthine (Sigma-Aldrich, New Delhi, India), 2.5 μg/ml gentamicin (Sigma-Aldrich, New Delhi, India) and 0.5% albumin II (Gibco, MA, USA). Human erythrocyte type O^+^ was used to control the percentage of infected erythrocytes. For parasite cultivation, the parasites were propagated in a culture flask containing complete medium with 2% hematocrit, and the cultures were maintained in an incubator (Panasonic, Model; MCO-175, Japan) at 37 °C in an environment of 5% CO_2_.

#### Drug and extract preparation

Artesunate (Sigma-Aldrich, New Delhi, India) was used as a positive control in this study. Stock solutions of the drug and each extract were prepared by dissolving the substance in dimethyl sulfoxide (DMSO) (Merck, Darmstadt, Germany). Thereafter, two-fold dilutions from a starting concentration of 500 mg/ml were made to prepare the final concentration of the extract in the range of 1.22–625 μg/ml, while a dilution series of artesunate was diluted from a stock of 4 mg/ml.

A final concentration of DMSO of 0.5% was used for all tested concentrations of the extracts.

#### Assessment of antimalarial activity against *P. falciparum* using the pLDH method

The in vitro antimalarial activity was evaluated by the measurement of parasite lactate dehydrogenase (pLDH) activity [21]. Briefly, parasite suspensions were prepared in 2% hematocrit with a final parasitemia of 1% for drug testing. Then, the parasites were seeded into the cell culture plate (199 μl per well), followed by the addition of crude extract, artesunate or DMSO solutions (1 μl per well) to serve as the experimental tests and the positive and negative controls, respectively. The plates were incubated at 37 °C in 5% CO_2_. After 48 h, the erythrocytes in culture plates were hemolyzed by freeze-thaw cycles: the erythrocytes were frozen at −80 °C for 30 min and thawed at 37 °C in a water bath for 30 min. This cycle was repeated three times to ensure that all erythrocytes were hemolyzed. This step is performed to release the cell content, including pLDH, when erythrocytes rupture [22]. Malstate reagent (100 μl per well) and nitroblue tetrazolium/phenazine ethosulfate solution (20 μl per well) (Calbiochem, Sigma-Aldrich, New Delhi, India) were mixed with 20 μl of lysate-infected red cells. Then, the plates were incubated in the dark at room temperature for 60 min. The results were assessed by measuring the optical density at 650 nm using a microplate reader (Biotek Eon, USA). The assays were performed in duplicate. Finally, the half maximal inhibitory concentration (IC_50_) of each sample was calculated in GraphPad Prism version 6; the data are presented as the mean ± standard deviation (SD). Briefly, after OD measurement, the concentrations were transformed into log(x). Next, the sample ODs were normalized to a percentage scale. Then, log(inhibitor) vs. normalized response-variable slope was used to calculate the IC_50_.

#### Assessment of cytotoxicity using the MTT method

The 3-(4,5-dimethythiazol-2-yl)-2,5-diphenyl tetrazolium bromide (MTT) colorimetric method was used to evaluate the cytotoxicity of the extract in Vero cells (Elabscience, Wuhan, Hubei, China). Dulbecco’s modified Eagle’s medium (CaissonLab, Smithfield, UT, USA) supplemented with 10% fetal bovine serum (CaissonLab, Smithfield, UT, USA) was used for Vero cell culture. The crude extract was dissolved in DMSO to give a stock solution of 1 mg/ml. The final concentrations of the extract were prepared in the range of 5–80 μg/ml. Doxorubicin (Sigma-Aldrich, New Delhi, India) was used to induce cell toxicity as a positive control. Briefly, 10^4^ cells/ml were seeded in a cell culture plate (100 μl/well) and then incubated at 37 °C in 5% CO_2_ for 24 h. Thereafter, 100 μl of different test compounds was added, and the test plates were incubated at 37 °C in 5% CO_2_ for 48 h. MTT reagent was then added to each well (50 μl/well), and the test plates were incubated at 37 °C for 2 h. The supernatant was removed, and 100 μl of DMSO was added to each well to solubilize the formazan crystals. The absorbance was read at 590 nm using a microplate reader (Biotek Eon, USA). The assays were performed in duplicate. The percentage growth inhibition was calculated to determine the cytotoxicity. The data are expressed by 50% cytotoxicity concentration (CC_50_) as the mean ± SD using GraphPad Prism version 6. Briefly, after OD measurement, the concentrations were transformed into log(x). Next, the sample ODs were normalized to a percentage scale. Then, log(inhibitor) vs. normalized response-variable slope was used to calculate the CC_50_.

### In vivo assay

#### Animals and parasites

Twenty-five male and fifteen male ICR mice weighing 20–30 g were obtained from Nomura Siam International Co., Ltd., Pathumwan, Bangkok, Thailand. Mice were housed in laboratory conditions (animal room: 22 °C (± 3 °C), humidity: 50–60%) for 7 days and allowed standard pelleted feed and water *ad libitum*. *Plasmodium berghei* strains ANKA, MRA-311 contributed by Thomas F. McCutchan were obtained through the Biodefense and Emerging Infections Research Resources Repository (BEI Resources).

#### 4-day suppressive test

An extract with potent antimalarial activity and nontoxicity in vitro was further evaluated for its effect against *P. berghei*-infected mice using a 4-day suppressive test. The test was performed according to the method described by Knight and Peters [23], with some modifications. Twenty-five male mice were randomly divided into five groups of five mice each. Three groups were assigned to the extract-treated groups, in which mice were administered daily oral doses of ethanolic wood extract of *M. fragrans* at 200, 400 and 600 mg/kg body weight as low, moderate and high doses, respectively, according to previous studies [17, 24, 25]. Two control groups were assigned: the positive control group was treated with 6 mg/kg artesunate, while the negative control group was given an equivalent volume of 7% Tween 80 and 3% ethanol in distilled water. On the first day, mice were inoculated with 200 μl of 1 × 10^7^ *P. berghei-*infected erythrocytes by intraperitoneal injection. Subsequently, each treated group received the ethanolic wood extract of *M. fragrans* 4 h after infection, which then continued daily for 4 days. In addition, the changes in body weights of each mouse in all groups were recorded before infection on day 0 and after treatment on day 4 using a sensitive digital balance (Mettler Toledo, Switzerland). On the fifth day, blood was collected by cutting the tip of the mouse tail to prepare thin blood films and to determine parasitemia. The mice were sacrificed by sodium pentobarbital overdose (120 mg/kg intraperitoneal injection). The slides were stained with 10% Giemsa solution to reveal infected cells under a light microscope (×100 oil immersion, Olympus CX31, Model CX31RBSFA, Japan). Finally, the percentage suppression of parasitemia was determined as follows:

$$\%\kern0.5em \mathrm{Suppression}=\frac{\mathrm{A}\hbox{-} \mathrm{B}}{\mathrm{A}}\times 100$$

where A is the mean percentage of parasitemia in the negative control group, and B is the mean percentage of parasitemia in the extract-treated group.

#### Acute toxicity test

Male mice were randomly divided into three groups of five mice per group. Group one was the untreated control mice. Group two was given 7% Tween 80 solution, which was used as a negative control. Group three included mice treated with a single oral dose of 2000 mg/kg extract. The test was carried out according to the 2008 OECD guidelines [26]. The mice were fasted for 3 h before administering the ethanolic wood extract of *M. fragrans* by oral gavage. Thereafter, mice were observed after dosing for the first 30 min and then monitored for abnormal signs such as diarrhea, sleep, rigidity, depression, abnormal secretion and hair erection every 1 h, intermittently for 4 h, over a period of 24 h and then once daily for a total of 14 days. On day 14, the mice were anesthetized with intraperitoneal injection of 60 mg/kg sodium pentobarbital. After anaesthetization, blood was collected by cardiac puncture for biochemical analysis, and the liver and kidney tissues were removed for histopathological examination.

#### Biochemical analysis

In the acute toxicity test, blood samples were collected into heparinized tubes from mice by cardiac puncture. Plasma samples were obtained by centrifugation at 3000 rpm for 5 min, and then, the liver and kidney functions of the samples were evaluated using an AU480 chemistry analyzer (Beckman Coulter, Inc., USA). The activities of the following liver enzymes were analyzed: alanine aminotransferase (ALT), aspartate aminotransferase (AST), and alkaline phosphatase (ALP). Kidney function tests included blood urea nitrogen (BUN) and creatinine levels.

#### Histopathological study

Histopathological assessments were carried out according to a previous method [17]. Briefly, the liver and kidney were removed by dissection of the peritoneal cavity after blood collection. Tissues were fixed in 10% formalin buffer and then dehydrated with ethanol (70, 90, 96 and 100%). After fixation, the tissues were embedded in paraffin, and then, the tissue sections were stained with hematoxylin-eosin (H&E) and inspected under a light microscope (Olympus CX31, Model CX31RBSFA, Japan) for histopathological changes.

### Statistical analysis

The data in this study are expressed as the mean ± SEM**.** SPSS for Microsoft Windows version 17.0 (SPSS, IL, USA) was used to calculate normal distribution and analysis of variance (ANOVA). Significant differences among the groups were determined at the 5% significance level (*p* < 0.05).

## Results

### Percentage yield of crude plant material

The extraction yields for the aqueous and ethanolic extracts are shown in Table 2. The maximum percent yield was obtained with the ethanolic extract of *S. rarak* (30.23%), followed by the ethanolic extract of *D. loureiri* (20.73%) and the aqueous extract of *E. rheedii* (19.93%). The aqueous extract of *S. pinnata* showed a minimum percent yield of 1.91%.

**Table 2:** The yield percentages of the aqueous and ethanolic crude extracts of medicinal plants in the Mahanil-Tang-Thong formulation

Plant species	Yield (%)	
	Aqueous extract	Ethanolic extract
*Calamus axillaris* Becc*.*	2.11	3.52
*Dracaena loureiri* Gagnep.	12.44	20.73
*Entada rheedii* Spreng.	19.93	4.50
*Myristica fragrans* Houtt.	2.22	5.75
*Pogostemon cablin* (Blanco)	2.07	3.45
*Sapindus rarak* DC.	18.14	30.23
*Spondias pinnata* (L.f.) Kurz	1.91	3.18
*Tectona grandis* L.f.	2.65	4.42
*Tiliacora triandra* (Colebr.) Diels	2.80	4.67

### Phytochemical screening results

The results of phytochemical screening for secondary metabolites revealed that the medicinal plants in the Mahanil-Tang-Thong formulation contained a predominance of flavonoids, terpenoids, alkaloids, tannins and saponins (Table 3).

**Table 3:** Phytochemical constituents of the crude extracts of medicinal plants in the Mahanil-Tang-Thong formulation

Medicinal plants	Extract	Phytochemical constituents							
		FL	TP	AL	TN	AN	CG	SN	CR
*Calamus axillaris* Becc.	Ethanolic	+	+	–	+	–	–	++	+
	Aqueous	+	–	+	+	–	–	++	+
*Dracaena loureiri* Gagnep.	Ethanolic	++	+	–	–	–	–	–	+
	Aqueous	++	+	–	+	–	–	–	+
*Entada rheedii* Spreng.	Ethanolic	–	+	+	++	–	–	–	–
	Aqueous	–	–	–	+	–	–	++	+
*Myristica fragrans* Houtt.	Ethanolic	–	+++	–	–	–	–	–	+
	Aqueous	++	+	++	+	–	–	+	+
*Pogostemon cablin* (Blanco)	Ethanolic	+	–	++	++	–	–	+	+
	Aqueous	–	–	++	++	–	–	+	–
*Sapindus rarak* DC.	Ethanolic	–	–	+	–	–	–	+++	–
	Aqueous	+	–	–	+	–	–	+	+
*Spondias pinnata* (L.f.) Kurz	Ethanolic	–	+	–	+	–	–	–	+
	Aqueous	–	+	+	++	–	–	–	+
*Tectona grandis* L.f.	Ethanolic	+	–	–	–	–	–	–	–
	Aqueous	+	–	+	++	–	–	++	+
*Tiliacora triandra* (Colebr.) Diels	Ethanolic	–	–	–	+	–	–	+	–
	Aqueous	++	–	+++	+	–	–	+	

+++, Strong presence; ++, moderate presence; +, slight presence; −, absence

*FL* Flavonoid, *TP* Terpenoid, *AL* Alkaloids, *TN* Tannin, *AN* Anthraquinone, *CG* Cardiac glycosides, *SN* Saponins, *CR* coumarin

### pLDH antimalarial activity results

Eighteen different crude extracts were evaluated for antimalarial activity according to the pLDH method. The in vitro activity that inhibited half of the parasite growth (IC_50_) of each extract is shown in Table 4. According to antimalarial classification [27], high activity was defined as an IC_50_ of less than 5 μg/ml, promising activity at an IC_50_ of 5–15 μg/ml, moderate activity at 15–50 μg/ml and no activity at more than 50 μg/ml. Among the crude extracts tested, eight exhibited antimalarial activity against *P. falciparum.* Ethanolic extracts of *S. pinnata* and *T. grandis* showed high activity, with IC_50_ values of 2.46 and 3.21 μg/ml, respectively. Promising activity against the parasite was observed for the ethanolic extracts of *M. fragrans* (IC_50_ = 8.87 μg/ml) and *D. loureiri* (IC_50_ = 10.47 μg/ml). Moderate parasite growth inhibition activity was found for the ethanolic extract of *C. axillaris* and *P. cablin* and the aqueous extract of *M. fragrans* and *S. pinnata*, with IC_50_ values of 22.32, 24.49, 46.36 and 49.94 μg/ml, respectively. The ethanolic extract of the formulation exhibited moderate activity with an IC_50_ value of 37.63 μg/ml while its aqueous extract had no activity (IC_50_ = 100.49 μg/ml).

**Table 4:** In vitro antimalarial activity and cytotoxicity of medicinal plants in the Mahanil-Tang-Thong formulation

Medicinal Plant	Extract	pLDH: IC_50_ (μg/ml)	MTT: CC_50_ (μg/ml)
*Calamus axillaris* Becc.	Ethanolic	22.32 ± 0.00	54.69 ± 2.96
	Aqueous	52.37 ± 0.05	>80
*Dracaena loureiri* Gagnep.	Ethanolic	10.47 ± 0.00	55.67 ± 3.28
	Aqueous	104.00 ± 0.00	>80
*Entada rheedii* Spreng.	Ethanolic	365.40 ± 0.02	>80
	Aqueous	203.60 ± 0.02	42.64 ± 0.14
*Myristica fragrans* Houtt.	Ethanolic	8.87 ± 0.00	>80
	Aqueous	46.36 ± 0.01	>80
*Pogostemon cablin* (Blanco)	Ethanolic	24.49 ± 0.01	>80
	Aqueous	549.30 ± 0.07	>80
*Sapindus rarak* DC.	Ethanolic	75.76 ± 0.00	41.11 ± 1.13
	Aqueous	90.84 ± 0.02	43.52 ± 2.06
*Spondias pinnata* (L.f.) Kurz	Ethanolic	2.46 ± 0.69	48.03 ± 0.04
	Aqueous	49.94 ± 0.00	>80
*Tectona grandis* L.f.	Ethanolic	3.21 ± 0.00	29.11 ± 1.10
	Aqueous	120.00 ± 0.03	>80
*Tiliacora triandra* (Colebr.) Diels	Ethanolic	116.50 ± 0.01	>80
	Aqueous	117.00 ± 0.02	>80
Mahanil-Tang-Thong formulation	Ethanolic	37.63 ± 1.05	28.50 ± 2.39
	Aqueous	100.49 ± 1.05	250.95 ± 5.25
Artesunate	–	1.28 ± 0.71	ND
Doxorubicin	–	ND	1.23 ± 0.16

*ND* Not determined

### Cytotoxicity results

The measurement of cytotoxicity was expressed as the half-maximal cytotoxic concentration (CC_50_), that is, the concentration at which a 50% reduction in cell viability occurred. Table 4 shows the cytotoxic results of the extracts. According to the classification of cytotoxicity from a previous study [28], thirteen extracts exhibited nontoxicity to Vero cells, with CC_50_ values greater than 50 μg/ml. Five extracts presented moderate toxicity, with CC_50_ values ranging from 29.11 to 48.03 μg/ml.

The extract with IC_50_ values less than 15 μg/ml represents the candidate extract in the first step of searching for new antimalarial plant extracts [29]. Among the eighteen extracts tested in the in vitro pLDH assay, four ethanolic extracts were promising candidates: *S. pinnata*, *T. grandis*, *M. fragrans* and *D. loureiri*. When the candidates were evaluated for cytotoxicity against Vero cells, the extracts from *M. fragrans* and *D. loureiri* exhibited potential nontoxicity, with CC_50_ values greater than 80 μg/ml and 55.67 ± 3.28 μg/ml, respectively [28]. Based on the potency and toxicity, the ethanolic wood extract of *M. fragrans* was selected for the in vivo study because it possessed strong antimalarial activity and was nontoxic to normal cells.

### Four-day suppressive test results

The in vivo antimalarial activity in mice treated with the ethanolic wood extract of *M. fragrans* was evaluated, as shown in Table 5. The extract exhibited dose-dependent suppressive activity at doses of 200, 400 and 600 mg/kg, with suppressive effects of 8.59, 31.00 and 52.58%, respectively, with a median effective dose (ED_50_) of 574.20 ± 13.71 mg/kg. The comparison of the percentage of parasitemia showed the lowest value in mice treated with artesunate (2.56%). The percentage of parasitemia in mice treated with the extract at 400 and 600 mg/kg doses revealed significant differences (*p* < 0.05) compared to the negative control mice. However, mice treated with 200 mg/kg extract were not significantly different from the negative control group. Regarding the percentage change in body weight, a dose of 600 mg/kg body weight significantly prevented body weight loss compared to the negative control group (Table 6).

**Table 5:** Parasitemia suppressive activity of the ethanolic wood extract of *M. fragrans*

Group	Dose (mg/ml)	% Parasitemia	% Suppression
Negative control group	–	64.25 ± 0.57^b, d, e^	–
Artesunate	6	2.56 ± 0.83^a, c, d, e^	96.02 ± 1.35
Extract	200	58.73 ± 0.72^b, d, e^	8.59 ± 1.12
	400	44.33 ± 0.85^a, b, c, e^	31.00 ± 1.32
	600	30.47 ± 0.87^a, b, c, d^	52.58 ± 1.35

The data are presented as the mean ± SEM (*n* = 5 per group)

^a^Compared to negative control

^b^Compared to artesunate

^c^Compared to 200 mg/kg extract

^d^Compared to 400 mg/kg extract

^e^Compared to 600 mg/kg extract, *p* < 0.05

**Table 6:** Body weight change in the 4-day suppressive test of the ethanolic wood extract of *M. fragrans*

Group	Dose (mg/kg)	Day 0 (g)	Day 4 (g)	Change (%)
Negative control group	–	36.56 ± 1.25	35.17 ± 1.23	−3.82 ± 0.73^b, d, e^
Artesunate	6	35.95 ± 2.02	38.25 ± 2.07	6.42 ± 1.61^a, c, d, e^
Extract	200	35.91 ± 2.54	33.96 ± 2.31	−5.39 ± 1.50^b, d, e^
	400	35.23 ± 2.49	34.52 ± 2.81	−2.06 ± 1.69^a, b, c^
	600	34.23 ± 1.40	33.93 ± 1.35	−0.89 ± 0.18^a, b, c^

The data are presented as the mean ± SEM (*n* = 5 per group)

^a^Compared to negative control

^b^Compared to artesunate

^c^Compared to 200 mg/kg extract

^d^Compared to 400 mg/kg extract

^e^Compared to 600 mg/kg extract, *p* < 0.05

### Acute toxicity test results

The acute toxicity of ethanolic wood extract of *M. fragrans* was determined by physical and behavioral observation, liver-kidney function tests and histopathological analysis of treated mice. In the case of observations, administration of the extract with a single oral dose of 2000 mg/kg did not induce mortality during the 14-day observational period. Thus, the LD_50_ value (the concentration causing death in 50% of treated mice) was estimated to be greater than 2000 mg/kg body weight. General behavior, physiological activities or signs of toxicity, including changes in the color of skin, eyes, and urine, diarrhea, sleep, rigidity, depression, abnormal secretion and hair erection, were not observed during the experimental period.

### Biochemical analysis results

Biochemical changes in liver and kidney functions are presented in Table 7. The results of the kidney test were not significantly different from those of the controls. The levels of the liver enzymes AST and ALP in mice treated with the extract and the negative control group (7% Tween 80) were significantly increased compared to those of the infected untreated group. The levels of ALT between the treated and untreated groups were not significantly different.

**Table 7:** Biochemical profile of liver and kidney functions of the infected untreated group and the group treated with the ethanolic extract of *M. fragrans*

**Group**	**Liver function test**		
	**AST (U/l)**	**ALT (U/l)**	**ALP (U/l)**
Untreated group	67.00 ± 5.43	27.80 ± 6.53	83.26 ± 3.18
7% Tween 80	84.40 ± 1.82 ^a^	27.00 ± 2.00	72.30 ± 2.93
2000 mg/kg extract	92.60 ± 8.14 ^a^	24.80 ± 2.28	110.22 ± 19.30 ^a, b^
	**Kidney function test**		
	**BUN (mg/dl)**		**Creatinine (mg/dl)**
Untreated group	20.42 ± 1.71		0.53 ± 0.03
7% Tween 80	19.22 ± 1.69		0.56 ± 0.02
2000 mg/kg extract	21.64 ± 0.59		0.56 ± 0.03

^a^Significantly different from the untreated group

^b^Significantly different from 7% Tween 80 (negative control group) (*p* < 0.05)

### Histopathological examination results

**Fig. 1:**
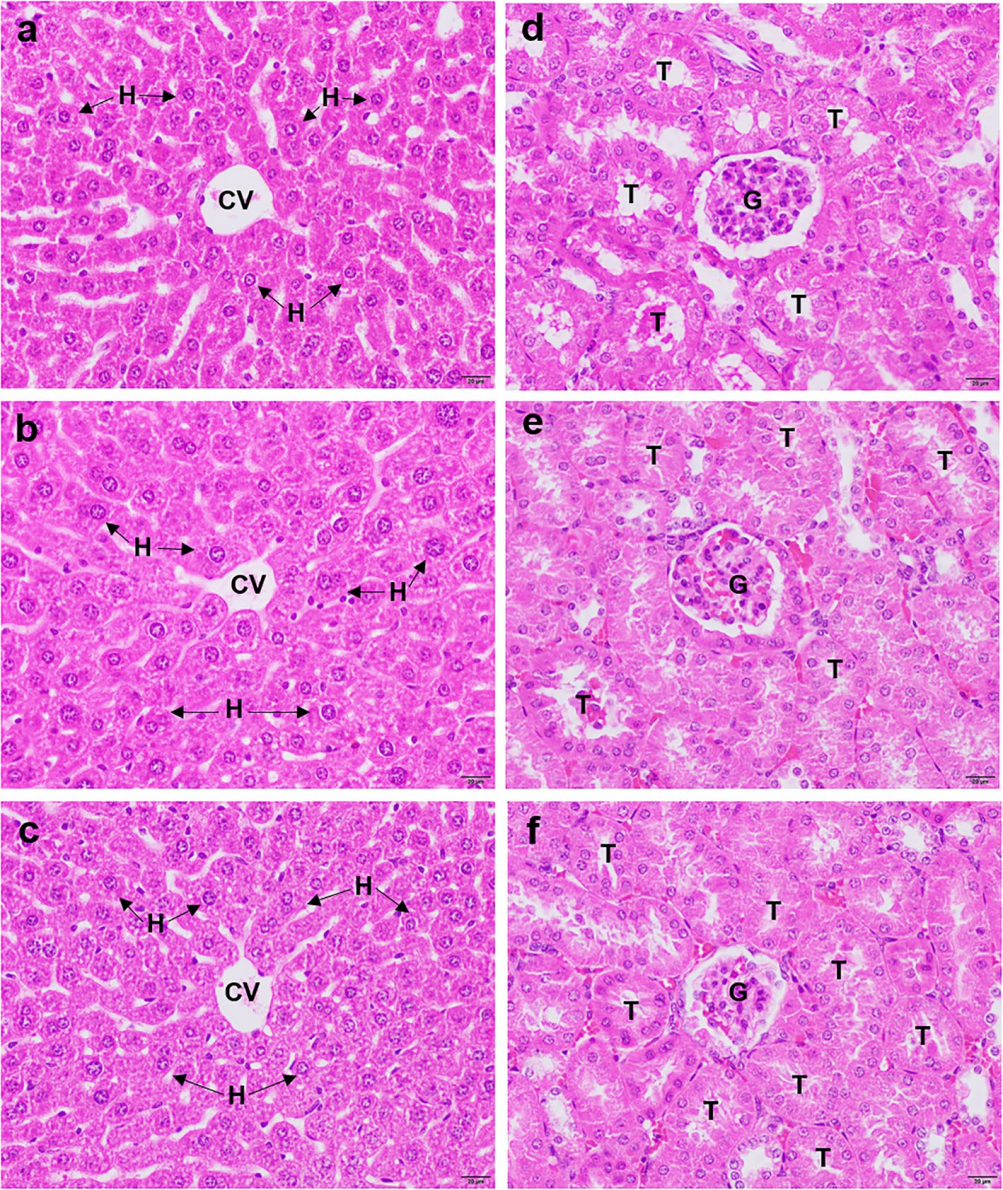
Histopathological study of liver and kidney tissues. Representative image of H&E staining of the liver and kidneys from the untreated group (**a**) and (**d**), the negative control group (**b**) and (**e**) and the extract-treated groups treated with 2000 mg/kg ethanolic *M. fragrans* extract (**c**) and (**f**). All images are 400× magnification. Bars, 20 μm; details showing (T), tubules, (G), glomerulus, (CV), central vein, and (H), hepatocyte

The histological changes in liver and kidney tissues were observed using a light microscope, as shown in Fig. 1. The histopathological examination revealed that liver sections of the negative control group (7% Tween 80) (Fig. 1b) and extract-treated groups (Fig. 1c) exhibited normal architecture of liver tissue compared to the untreated group (Fig. 1a). In addition, compared to the untreated group (Fig. 1d), the negative control group (7% Tween 80) (Fig. 1e) and extract-treated groups (Fig. 1f) demonstrated normal histology of kidney tissue, consisting of unchanged glomeruli and renal tubules.

## Discussion

The extract with IC_50_ values less than 15 μg/ml represents the candidate extract in the first step of searching for new antimalarial plant extracts [29]. Among the eighteen extracts tested in the in vitro pLDH assay, four ethanolic extracts were promising candidates: *S. pinnata*, *T. grandis*, *M. fragrans* and *D. Loureirin* that more potent than the formulation. When the candidates were evaluated for cytotoxicity against Vero cells, the extracts from *M. fragrans* and *D. loureiri* exhibited potential nontoxicity, with CC_50_ values greater than 80 μg/ml and 55.67 ± 3.28 μg/ml, respectively [28]. Based on the potency and toxicity, the ethanolic wood extract of *M. fragrans* was selected for the in vivo study because it possessed strong antimalarial activity and was nontoxic to normal cells. The current study was similar to a previous study showing that ethanolic seed extracts of *M. fragrans* and *D. loureiri* had potent antimalarial activity with IC_50_ values ranging from 4.6 to 9.9 μg/ml against chloroquine-resistant K1 and chloroquine-sensitive 3D7 clones of *P. falciparum* [16]. This study on in vitro antimalaria activity and cytotoxicity had the following limitations. To confirm the results, in vitro screening of antimalarial activity needs further evaluation via different methods with high sensitivity, such as the quantification of the parasite proteins histidine-rich protein 2 (HRP2) and lactate dehydrogenase (LDH) by enzyme-linked immunosorbent assay (ELISA) and DNA dye intercalation assays. In addition, additional strains of *P. falciparum* should be used for in vitro assays. For in vitro cytotoxicity testing, the extracts should be evaluated against other mammalian cell lines, such as HepG2 or HEK 293 cells, in future work.

The 4-day suppressive test is the most widely used assay for the preliminary screening of new antimalarial plant extracts [23, 30]. Moreover, body weight loss is one of the key characteristics and dramatic manifestations of malaria-infected mice [31]. The *M. fragrans* wood extract suppressed the percentage of parasitemia in a dose-dependent manner in early malaria infection. The highest percentage of parasitemia suppression was obtained at the dose of 600 mg/kg extract (52.58%). The lower suppression in the extract-treated groups than in the artesunate-treated group (96.02%), a positive control, might be due to pharmacokinetic and pharmacodynamic limitations, including low selectivity, poor oral bioavailability and first-pass metabolism of the extract [32].

Treatment of infected mice with the extract reduced body weight loss in a dose-dependent manner, and 600 mg/kg significantly prevented weight loss compared to that in the negative control group. Thus, these findings imply that 600 mg/kg *M. fragrans* extract could have the potential to reduce malaria infection. Although the active compound has yet to be identified in this study, the results from phytochemical analysis can be used to predict the possible antimalarial activity of a plant [33]. The ethanolic wood extract of *M. fragrans* had terpenoids and coumarins; thus, the antimalarial effect of *M. fragrans* in this study may be attributed to the single or combined effect of these compounds.

Regarding terpenoids, these compounds might interfere with the parasite’s polyisoprenoid protein biosynthesis, which occurs during the asexual intraerythrocytic developmental cycle [34]. In addition, the results of an acute toxicity study indicated that mice receiving the extract at a maximum dose of 2000 mg/kg did not show any signs of toxicity or mortality; as a result, the LD_50_ of the *M. fragrans* extract was estimated to be greater than 2000 mg/kg body weight, which is generally considered safe [35]. The safety of this plant is consistent with a previous report demonstrating that a methanolic kernel extract of *M. fragrans* exhibited hepatoprotective effects [36]. A wide variety of xenobiotics accumulate in the liver and kidneys, and most substances are biotransformed in the liver. Therefore, the assessment of liver and kidney function is essential in evaluating the toxicity of drugs and plant extracts [37, 38]. In fact, AST and ALT are biomarkers of liver integrity, but the ALT level is more specific to liver function than AST because AST is also found in abundance in kidneys, cardiac skeletal muscles and testes [35, 39]. Moreover, the degree of hepatic ALP alteration is used to assess the integrity of the bile duct epithelium [40].

In our study, the liver function parameters showed that the ethanolic wood extract of *M. fragrans* significantly altered the AST and ALP values. These findings may indicate that an extract can induce some degree of liver changes, which may result from the detoxification of compounds in this organ [41]. However, the ALT level was normal, and this value is a marker for monitoring acute drug-induced liver injury in early clinical trials [42]. The important markers of kidney function are creatinine and BUN [43]. The results regarding the kidneys, including biochemical analysis of kidney function and histopathological changes, were not different from those of control mice. Thus, this finding suggests that the extract has no harmful effect on the kidneys. Biochemical parameters were used as indices to monitor the severity of malaria. Liver enzyme activities are important indicators of hepatic dysfunction in patients with *P. falciparum* infection [44]. Acute *falciparum* malaria infection is associated with an increase in the serum activity of AST, ALT, and ALP [45]. In contrast, in a study on the general population and pregnant malaria patients, the activities of AST, ALP and total protein were elevated with a reduction in ALT, albumin and total bilirubin. The degree of alterations in these biochemical parameters depends on the level of parasitemia, nutritional status, malaria immunity and endemicity of the disease [46].

## Conclusion

The ethanolic wood extract of *M. fragrans*, a medicinal plant of the Mahanil-Tang-Thong formulation, exhibited potent antimalarial activity against *P. falciparum* and promising antimalarial activity in mice infected with chloroquine-sensitive *P. berghei*. This study reports for the first time the in vivo antimalarial activity of *M. fragrans*. Consequently, further work should be performed to identify active compounds from this plant to search for antimalarial drug candidates.

## Data Availability

The data associated with this study are included in this published article. Additional files are available from the corresponding author upon reasonable request.

## References

[1] Antony HA, Parija SC (2016). Antimalarial drug resistance: An overview. Trop Parasitol.

[2] Travassos MA, Laufer MK (2009). Resistance to antimalarial drugs: molecular, pharmacologic, and clinical considerations. Pediatr Res.

[3] Roper C, Pearce R, Bredenkamp B, Gumede J, Drakeley C, Mosha F (2003). Antifolate antimalarial resistance in southeast Africa: a population-based analysis. Lancet (London, England).

[4] Nair S, Williams JT, Brockman A, Paiphun L, Mayxay M, Newton PN (2003). A selective sweep driven by pyrimethamine treatment in southeast asian malaria parasites. Mol Biol Evol.

[5] Wellems TE, Plowe CV (2001). Chloroquine-resistant malaria. J Infect Dis.

[6] Tse EG, Korsik M, Todd MH (2019). The past, present and future of anti-malarial medicines. Malar J.

[7] Andrews KA, Wesche D, McCarthy J, Möhrle JJ, Tarning J, Phillips L (2018). Model-informed drug development for malaria therapeutics. Annu Rev Pharmacol Toxicol.

[8] Ashley EA, Dhorda M, Fairhurst RM, Amaratunga C, Lim P, Suon S (2014). Spread of artemisinin resistance in *Plasmodium falciparum* malaria. N Engl J Med.

[9] Menard D, Dondorp A (2017). Antimalarial drug resistance: a threat to malaria elimination. Cold Spring Harb Perspect Med.

[10] Pulcini S, Staines HM, Lee AH, Shafik SH, Bouyer G, Moore CM (2015). Mutations in the *Plasmodium falciparum* chloroquine resistance transporter, *Pf*CRT, enlarge the parasite's food vacuole and alter drug sensitivities. Sci Rep.

[11] Gregson A, Plowe CV (2005). Mechanisms of resistance of malaria parasites to antifolates. Pharmacol Rev.

[12] Isozumi R, Uemura H, Kimata I, Ichinose Y, Logedi J, Omar A (2015). Novel mutations in K13 propeller gene of artemisinin-resistant *Plasmodium falciparum*. Emerg Infect Dis.

[13] Tajuddeen N, Van Heerden FR (2019). Antiplasmodial natural products: an update. Malar J.

[14] Royal Thai Government Gazette. National list of essential medicines 2020 [Internet]. 2020 [cited 2021 Jan 2]. Available from: http://dmsic.moph.go.th/index/detail/8392.

[15] Ashley EA, Pyae Phyo A, Woodrow CJ (2018). Malaria. Lancet.

[16] Thiengsusuk A, Chaijaroenkul W, Na-Bangchang K (2013). Antimalarial activities of medicinal plants and herbal formulations used in Thai traditional medicine. Parasitol Res.

[17] Chaniad P, Techarang T, Phuwajaroanpong A, Punsawad C (2019). Antimalarial activity and toxicological assessment of *Betula alnoides* extract against *Plasmodium berghei* infections in mice. Evid Based Complement Alternat Med.

[18] Ngbolua K-T-N (2014). Phytochemical screening of some medicinal plants traditionally used by African women in Kinshasa city (DR Congo) for their intimate hygiene and evaluation of the pH of derived recipes. J Modern Drug Discov Drug Deliv Res.

[19] Malar G, Chinnachamy C (2017). Phytochemical screening, total flavonoid, total terpenoid and anti-inflammatory activity of aqueous stem extract of *Salacia oblonga*. J Chem Pharm.

[20] Trager W, Jensen JB (2005). Human malaria parasites in continuous culture. 1976. J Parasitol.

[21] Makler MT, Hinrichs DJ (1993). Measurement of the lactate dehydrogenase activity of *Plasmodium falciparum* as an assessment of parasitemia. Am J Trop Med Hyg.

[22] Zofou D, Tene M, Ngemenya MN, Tane P, Titanji VP (2011). *In vitro* antiplasmodial activity and cytotoxicity of extracts of selected medicinal plants used by traditional healers of Western cameroon. Malar Res Treat.

[23] Peters W, Portus H, Robinson L (1995). The four-day suppressive *in vivo* antimalarial test. Ann Trop Med Parasitol.

[24] Muluye AB, Desta AG, Abate SK, Dano GT (2019). Anti-malarial activity of the root extract of *Euphorbia abyssinica* (Euphorbiaceae) against *Plasmodium berghei* infection in mice. Malar J.

[25] Misganaw D, Engidawork E, Nedi T (2019). Evaluation of the anti-malarial activity of crude extract and solvent fractions of the leaves of *Olea europaea* (Oleaceae) in mice. BMC Complement Altern Med.

[26] OECD (2008). Test No. 425: Acute Oral Toxicity: Up-and-Down Procedure.

[27] Lusakibanza M, Mesia G, Tona G, Karemere S, Lukuka A, Tits M (2010). *In vitro* and *in vivo* antimalarial and cytotoxic activity of five plants used in congolese traditional medicine. J Ethnopharmacol.

[28] Berthi W, González A, Rios A, Blair S, Cogollo Á, Pabón A (2018). Anti-plasmodial effect of plant extracts from *Picrolemma huber*i and *Picramnia latifolia*. Malar J.

[29] Kweyamba P, Zofou D, Efange N, Assob J, Kitau J, Nyindo M (2019). *In vitro* and *in vivo* studies on anti-malarial activity of *Commiphora africana* and *Dichrostachys cinerea* used by the Maasai in Arusha region, Tanzania. Malar J.

[30] Sherman I (1998). Malaria : parasite biology, pathogenesis, PRO.

[31] Basir R, Rahiman SF, Hasballah K, Chong W, Talib H, Yam M (2012). *Plasmodium berghei* ANKA infection in ICR mice as a model of cerebral malaria. Iran J Parasitol.

[32] Ebiloma G (2011). Suppressive, curative and prophylactic potentials of *Morinda lucida* (Benth) against erythrocytic stage of mice infective chloroquine sensitive *Plasmodium berghei* NK-65. Br J Appl Sci Technol.

[33] Shaikh J, Patil MK (2020). Qualitative tests for preliminary phytochemical screening: An overview. Int J Chem Stud.

[34] Gabriel H, Sussmann R, Kimura E, Rodriguez A, Bofill Verdaguer I, Leite G (2018). Terpenes as potential antimalarial drugs.

[35] Ugwah-Oguejiofor CJ, Okoli CO, Ugwah MO, Umaru ML, Ogbulie CS, Mshelia HE (2019). Acute and sub-acute toxicity of aqueous extract of aerial parts of *Caralluma dalzielii* N. E. Brown in mice and rats. Heliyon..

[36] Dkhil MA, Abdel Moneim AE, Hafez TA, Mubaraki MA, Mohamed WF, Thagfan FA (2019). *Myristica fragrans* kernels prevent paracetamol-induced hepatotoxicity by inducing anti-apoptotic genes and Nrf2/HO-1 pathway. Int J Mol Sci.

[37] Evans TJ, Peterson ME, Talcott PA (2013). Chapter 2 - toxicokinetics and toxicodynamics. Small Animal Toxicology.

[38] Abid R, Mahmood R (2018). Acute and sub-acute oral toxicity of ethanol extract of *Cassia fistula* fruit in male rats. Avicenna J Phytomed.

[39] Lawal B, Shittu OK, Oibiokpa FI, Mohammed H, Umar SI, Haruna GM (2016). Antimicrobial evaluation, acute and sub-acute toxicity studies of allium sativum. J Acute Dis.

[40] Giannini EG, Testa R, Savarino V (2005). Liver enzyme alteration: a guide for clinicians. CMAJ..

[41] Muia B, Mbaria J, Kanja LA, Gitahi N, Okumu P, Okumu M (2020). Acute and sub-acute toxicity study of the root extracts of *Fagaropsis hildebrandtii* in mice and evaluation of their antimicrobial effects. F1000Research.

[42] Ozer J, Ratner M, Shaw M, Bailey W, Schomaker S (2008). The current state of serum biomarkers of hepatotoxicity. Toxicology..

[43] Sood MM, Saeed M, Lim V, Cordova F, Komenda P, Malik A (2015). The urea-to-creatinine ratio is predictive of worsening kidney function in ambulatory heart failure patients. J Card Fail.

[44] Chikezie P, Opara R (2013). Serum lipid profile and hepatic dysfunction in moderate *Plasmodium falciparum* infection. J Public Health Epidemiol.

[45] Al-Salahy M, Shnawa B, Abed G, Mandour A, Al-Ezzi A (2016). Parasitaemia and its relation to hematological parameters and liver function among patients malaria in Abs, Hajjah, Northwest Yemen. Interdiscip Perspect Infect Dis.

[46] Adamu J (2019). Effects of malaria infection on some haematological and biochemical parameters in the general population and pregnant malaria patients attending two district hospitals in Niger State, Nigeria. Glob J Infect Dis Clin Res.

